# Associations between precarious employment trajectories and mental health among older workers in Germany: Vertical and horizontal inequalities

**DOI:** 10.5271/sjweh.4160

**Published:** 2024-05-01

**Authors:** Max Rohrbacher, Hans Martin Hasselhorn, Nuria Matilla-Santander

**Affiliations:** 1 Department of Occupational Health Science, School of Mechanical Engineering and Safety Engineering, University of Wuppertal, Wuppertal, Germany.; 2 Unit of Occupational Medicine, Institute of Environmental Medicine, Karolinska Institutet, Stockholm, Sweden.

**Keywords:** Group-based trajectory modelling, older employee, prospective cohort

## Abstract

**Objective:**

The aim of the study was to investigate the longitudinal association between multi-dimensionally measured precarious employment (PE) trajectories and mental health among older employees in Germany.

**Methods:**

Current data from the German lidA study was used, including panel cases, who participated in all four survey waves (2011, 2014, 2018, 2022). The study comprised 1636 subjects, aged 46 and 52 years at baseline. Group-based trajectory modelling was used to model PE trajectories based on a score combining multiple items from the dimensions employment insecurity and income inadequacy. The association between PE trajectories (2011–2022) and mental health (2022) was tested using weighted logistic regression.

**Results:**

We identified a PE trajectory with upward movement that best described 13.6% of the study sample. Representation in this group was socially unequally distributed with noticeably larger shares of female, lower-educated and lower-skilled workers in PE. Women following this trajectory had increased odds [odds ratio (OR) 1.68–1.82] of reporting poor mental health in 2022 compared to their counterparts in constant non-PE. This was not the case for men (OR 0.37–0.51).

**Conclusions:**

Our findings highlight horizontal and vertical inequalities with respect to exposure to and consequences of PE. Future labor market reforms should improve protection of women, who will likely be disadvantaged by accumulating employment-related mental health risks over the course of their lives.

Existing evidence clearly suggests adverse effects of precarious employment (PE) on mental health (1). There is growing agreement that PE is understood as an "accumulation of various unfavorable facets of employment quality" (2, p391), thus constituting a multi-dimensional construct regularly combining employment insecurity, income inadequacy and a lack of rights and protection (3).

During the past two decades, PE has received increased attention from both public and public health research. Different international research groups have laid important corner stones for future research on PE and its effects on health including: the conceptual model on the pathways between PE and health (4); Rönnblad et al’s (1) review of the current evidence of the effect of PE on mental health, which demonstrated a scarcity of high-quality prospective studies; a review of commonly used dimensions and definitions of PE (3); and recent high-quality register-based cohort studies (5, 6).

So far, most high-quality studies on the association between PE and mental health have derived from Sweden (eg 7, 8,). Previously, authors have highlighted that the exposure to health-adverse employment conditions is unequally distributed along vertical (eg, education or occupation) and horizontal (eg, sex or migrant status) social positions (eg 9–11,). The magnitude of these vertical and horizontal inequalities likely varies between countries, given that welfare states act as "institutional filters" with respect to exposure and susceptibility to PE – this may limit the transferability of existing findings to other countries (4, 9, 12, 13).

Thus, evidence from outside Scandinavian countries would add to the existing knowledge. Only few studies succeeding Rönnblad et al's review (1) have used multi-dimensional measurements of PE. These stem from Scandinavia (5, 14) and also Germany (15–17). Based on data from the German Study on Mental Health at Work (S-MGA), Demiral et al (16) found that a cumulative PE exposure index combining multiple PE indicators was significantly associated with the development of depressive symptoms during the 5-year follow-up among men, but not women, aged 31–60 years (16). In Pförtner et al's study (15), which is based on data from the German Socio-Economic Panel, both prolonged PE and upward and downward mobility were associated with poor mental health [Short Form-12 Health Survey (SF-12)] over a 16-year follow-up among persons aged 18–67 years of both sexes (however, stronger among men) (15). To our knowledge, these are the only studies longitudinally investigating the effects of a multi-dimensionally measured PE on mental health among workers in Germany.

Still, there are two design features of these two German studies that may be regarded as limitations: first, the wide age range of included subjects and, second, the limited number of follow-ups. Exposure to and experience of PE likely varies between different age groups given the insider-outsider logic of the German labor market. Those who are already employed and established in the labor market are called 'insiders' (often mid-career and older male workers), while labor market entrants and those with interrupted, non-continuous careers (more often women) may be regarded as 'outsiders' (9, 18). Moreover, the consequences of PE might differ in dependence of the workers' proximity to retirement and their financial needs (19). Thus, even when statistical analyses are adjusted for age, the inclusion of multiple age groups might obscure the age- and context-dependent strength of the association between PE and mental health. Therefore, a narrower age range might be a strength when investigating PE conditions and their effects on health. Secondly, only Pförtner et al (15) measured exposure to PE at more than one time point – namely two. Given the time-varying nature of employment relations and its quality throughout the (late) career and the cumulative effect of different PE dimensions, the assessment of PE at more/multiple time points (trajectories) may help to prevent misclassification of the exposure (12, 20).

To the knowledge of the authors, there is no German study investigating the association between multi-dimensionally measured PE trajectories and mental health among older workers (from the German baby boom generations). Our aim is to fill this research gap.

## Methods

**Figure 1:**
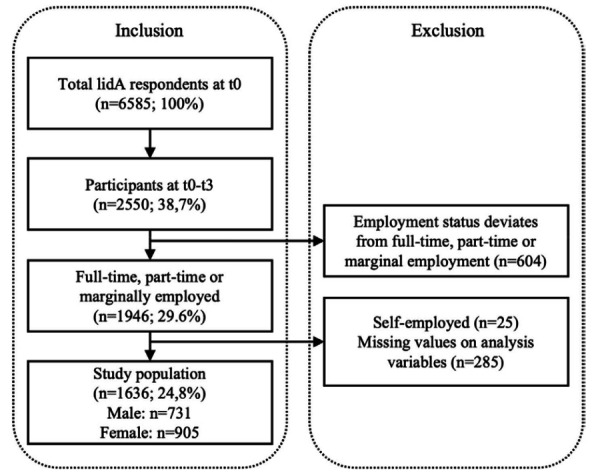
Flow chart of inclusion and exclusion criteria.

This study is based on data from the German lidA study, a prospective cohort study on the topics age, health and labor participation (21). lidA includes a representative sample of socially insured employees (initially excluding self-employed and sworn civil servants) from the German baby boom generation born in either 1959 or 1965 (21), sampled from the official process data on employment histories of the German Institute for Employment Research. Response rates are reported in the lidA method reports (22–25) and are similar to those of other employee surveys, eg, the S-MGA (26). Our analysis used data from subjects born either 1959 or 1965, aged 52 and 46, respectively, at baseline and followed up for 11 years. Subjects who participated in all study waves (t0=2011, t1=2014, t2=2018, t3=2022/2023 [referred to as 2022]) were eligible to be included in this study (N=2291). We excluded subjects, whose employment status deviated from full-time (≥35 hours/week), part-time, or marginal, ie, long-term sick, 'other' [eg, on (parental) leave], those in a qualification measure or unemployed and pensioners, and those who were self-employed in any of the waves (figure 1). Lastly, cases with missing information on analysis variables were excluded.

### Mental health (t_3_)

Mental health was assessed at t_3_ (outcome) and t_0_ (adjustment) using the mental component of the SF-12. Following Nübling et al's procedure (27), we created a Mental Component Summary (MCS) score ranging from 0 (lowest) to 100 (highest). For our analysis, we chose three different cut-offs to determine poor mental health: The first cut-off was set at 47.0, which corresponds to the 25^th^ percentile of the current sample at t_0_. Subjects with values of ≤47.0 were regarded as poor mental health cases. The second cut-off was set at 45.6 (21^st^ percentile), which Vilagut et al (28) suggested to be the optimal cut-off to detect 30-day depressive disorders in European samples. The third cut-off was set at 42.0 (14^th^ percentile), which Ware et al (29) recommended to be indicative of a clinical depression based on a US sample.

### Precarious employment trajectories (t_0_–t_3_)

We followed three steps when building the PE trajectories. First, we searched for the most suitable variables available at all four waves of the lidA survey data for a multi-dimensional measurement of PE. Our search was guided by Kreshpaj et al's article (3). From the three suggested dimensions – employment insecurity, income inadequacy and lack of rights and protection – only the first two could be operationalized with our data. Three items were used to cover the dimension employment insecurity, namely job threat (yes/no), temporary employment (yes/no) and multiple jobs (yes/no). To cover the dimension income inadequacy, we calculated a personal hourly net income based on information on monthly wage and the total amount of weekly working hours, following Demiral et al's descriptions (16). We then built five income groups (<60, 60–79, 80–99, 100–149, ≥150%) based on the median personal income in the study population. The calculation of the median and computation of income groups was done separately for each study wave to account for overall increases in income (eg, group <60% = income <€6.70/hour at t_0_ and <€8.90/hour at t_3_).

In a second step, we built a summative score following Jonsson et al (30). Job threat takes the values -2 (yes, job threat) and 0 (no job threat). Temporary employment takes the values -2 (temporary) and 0 (permanent). Multiple jobs take the values -1 (≥2 jobs) and 0 (1 job). The income level scores -2 (<60%), -1 (60–79%), 0 (80–99%), 1 (100–149%) and 2 (≥150%). The range of the resulting sum score of the four items was -7–2 (see supplementary material, www.sjweh.fi/article/4160, table S1).

In a third step, the PE trajectories were built by applying group-based trajectory modelling (GBTM) (31, 32). The model selection process was guided by several statistical criteria as well as subjective judgement/ domain knowledge (cf, 32). We specified a censored normal distribution for the PE score. The number of trajectory groups was determined based on the Bayesian Information Criterion (BIC) and Akaike Information Criterion (AIC) (see supplementary table S2). BIC and AIC closer to zero indicate a better model fit. We pre-determined a model choice set (cf, 32) of minimum three groups (PE, borderline PE, constant non-PE) and maximum six groups (constant PE, PE to non-PE, non-PE to PE, borderline PE, constant non-PE low, constant non-PE high). If only minor changes of BIC and AIC were observed between models, the most parsimonious model was selected (32). We chose a four-group option, since more groups lead to only marginal changes of the BIC and AIC and more groups in non-PE. Subsequently, the level of polynomials for each group trajectory was adjusted to achieve P<0.01 for the parameter estimate in the highest function (cf, 6). This resulted in a linear shape of all trajectories since P<0.01 could not be reached adding quadratic or cubic terms. The performance of the final model was assessed via the average posterior probability of assignment (≥0.7 for all groups), the odds of correct classification (>5.0 for all groups), the estimated group probabilities versus the proportion of the sample assigned to the group (close correspondence of both measures) and the 99% confidence intervals (CI) for group membership probability (reasonably narrow) (6, 32) (see supplementary table S3).

### Covariates

Based on a directed acyclic graph (DAG) (supplementary figure S4), we chose the following minimal sufficient adjustment set for estimating the total effect of PE trajectories (t_0_–t_3_) on mental health (t_3_): Age at baseline [46 (born 1965)/52 (born 1959)], sex (male/female), mental health (t_0_), migrant status (non-migrant/ 1^st^ and 2^nd^ generation migrant), partner status (t_0_) (partner / single), education (t_0_) and occupation (t_0_). The level of education was assessed with a score combining education and vocational training (33) and categorized into three classes of low, moderate, and high education (for more details see 33, p6). To measure a person's occupation, the German Blossfeld classification was used, consisting of 12 occupational categories based on KldB1988 (35). To reduce the number of categories, we classified these 12 occupations into manual and non-manual occupations and according to the degree of qualification following Götz et al (36), resulting in five groups (non-qualified manual, qualified manual; non-qualified non-manual; qualified non-manual, highly qualified non-manual).

### Statistical analysis

We first showed the trajectory groups identified by GBTM. Secondly, the sample characteristics were displayed using the PE trajectories as column variables and the socioeconomic variables as row variables. For all statistical analyses, the two constant non-PE groups were combined. For descriptive purposes, we displayed row percentages to highlight the relative fractions of socioeconomic groups within each PE group. Supplementary tables S5, S6 show these row percentages stratified by sex, supplementary table S7 shows column percentages, including the prevalence of poor mental health depending on cut-off. Next, we ran adjusted logistic regression analyses to obtain odds ratios (OR) and 95% CI for poor mental health in dependence of PE group membership. This was first done in the sample including women and men, adjusting for sex, then separately for women and men. We controlled for the minimal sufficient adjustment set. All main analyses were weighted by a longitudinal weight, which combines a post-stratification weight for t_0_ and a stabilized inverse probability weight to account for selection into the current sample including age, sex, education, migrant status and occupation as predictors (mean weighting factors in the supplementary tables S8 and S9).

### Sensitivity analysis

We conducted several additional and sensitivity analyses to check the robustness of our findings. First, we repeated the logistic regression analyses without using a sample weight. Second, we conducted ordinary least squares regression using SF-12 MCS as a continuous outcome, adjusting for the same set of covariates (including continuous instead of binary SF-12 MCS at t_0_). Furthermore, we provided cross-tables to display the distribution of PE components by PE groups for each wave, separately for men and women. Next, we repeated the GBTM stratified by sex to check how it would alter the trajectory group compositions. Lastly, we repeated the model building and logistic regression allowing for up to two unemployment spells over the second (t_1_) and third wave (t_2_). We still required subjects to be employed in the first (t_0_) and last (t_3_) wave to avoid confounding by unemployment on the follow-up mental health.

### Ethical approval

The Ethics Committee of the University of Wuppertal approved the protocol for the lidA Cohort study [5 December 2008 (Sch/Ei Hasselhorn) and 20 November 2017 (MS/BB 171025 Hasselhorn). All subjects included in the study provided verbal consent for their participation in waves 1–4 of the lidA cohort study.

## Results

**Figure 2:**
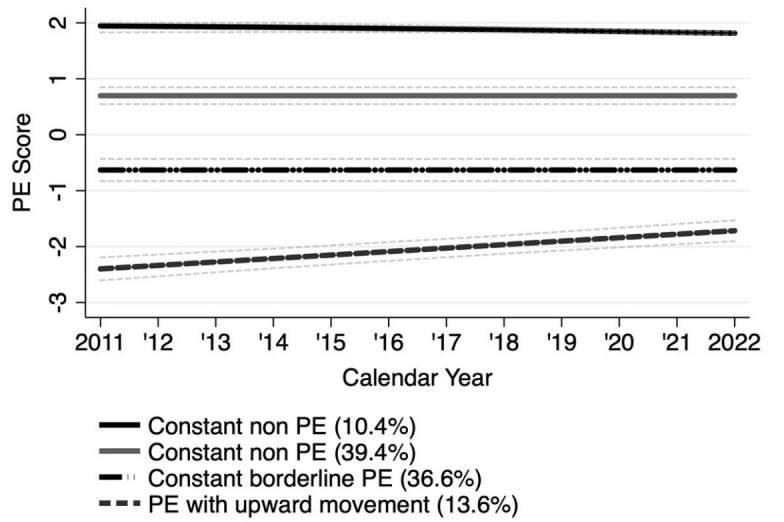
Trajectories of precarious and non-precarious employment (PE) (N=1636). An individual was regarded as precariously employed when the PE score was ≤-2 (% = proportion by posterior probability-based classification). Measurements took place in 2011, 2014, 2018, 2022.

Figure 2 shows the trajectory groups with 95% CIs. The percentages describe the proportions by posterior probability-based classification. The two upper lines show the two constant non-precarious trajectories. The black line shows the trajectory for 10.4% of the sample with values close to the maximum of 2. A second non-PE trajectory with values close to 1 described 39.4% of subjects. Next, a constant borderline PE trajectory was identified (36.6%). Lastly, the grey dotted line shows a PE trajectory with upward movement. The trajectory starts below a PE-Score of -2 and shows a slight upward movement over time. This group best described 13.6% of the sample.

In table 1 the proportions within each category of the socioeconomic variables that are represented in the trajectory groups are shown. 20.2% of female workers and 5.5% of male workers were in the PE trajectory with upward movement. Furthermore, 17.7% of the low educated workers, 14.9% of the moderately educated and 7.8% of high educated workers followed this PE trajectory. Similarly, remarkably larger shares of non-qualified workers followed this PE trajectory. This was true for both manually (t_0_: 18.9%; t_3_: 16.7%) and non-manually (t_0_: 33.6%; t_3_: 31.0%) employed persons.

**Table 1:** Sample characteristics. [PE=precarious employment].

		PE with upward movement (N=223)		Borderline PE (N=599)		Non-PE (N=814)
		N (%)		N (%)		N (%)
Age in years (t_0_)						
	46 (born 1965)	137 (13.3)		374 (36.4)		516 (50.2)
	52 (born 1959)	86 (14.1)		225 (36.9)		298 (48.9)
Sex						
	Male	40 (5.5)		208 (28.5)		483 (66.1)
	Female	183 (20.2)		391 (43.2)		331 (36.6)
Migrant						
	Non migrant	188 (13.3)		532 (37.6)		695 (49.1)
	Migrant	35 (15.8)		67 (30.3)		119 (53.8)
Educational level						
	Low	51 (17.7)		133 (46.2)		104 (36.1)
	Moderate	140 (14.9)		378 (40.3)		419 (44.7)
	High	32 (7.8)		88 (21.4)		291 (70.8)
Occupation (t_0_)						
	Non-qualified manual	21 (18.9)		51 (45.9)		39 (35.1)
	Qualified manual	24 (9.1)		90 (34.1)		150 (56.8)
	Non-qualified non-manual	81 (33.6)		120 (49.8)		40 (16.6)
	Qualified non-manual	88 (11.5)		306 (40.1)		369 (48.4)
	Highly qualified non-manual	9 (3.5)		32 (12.5)		216 (84.0)
Occupation (t_3_)						
	Non-qualified manual	17 (16.7)		46 (45.1)		39 (38.2)
	Qualified manual	24 (9.3)		87 (33.9)		146 (56.8)
	Non-qualified non-manual	80 (31.0)		130 (50.4)		48 (18.6)
	Qualified non-manual	96 (12.2)		308 (39.3)		380 (48.5)
	Highly qualified non-manual	6 (2.6)		28 (11.9)		201 (85.5)

Table 2 displays the results from the logistic regression analyses. The first column shows the results from the unstratified sample (additionally adjusted for sex), the second and third column show the findings from the sex-stratified analyses. The results from the unstratified analysis indicate increased odds to report poor mental health at t_3_ for the group of employees following the 'PE with upward movement' trajectory compared to those following a 'constant non-PE' trajectory. Regardless the selected cut-off this association was non-significant. Using the cut-off 47.0 for SF-12 MCS the OR was 1.22 (95% CI 0.80–1.86). Using the cut-offs 45.6 and 42.0, the OR were 1.37 (95% CI 0.90–2.09) and 1.47 (95% CI 0.92–2.34) respectively.

**Table 2:** Longitudinal association between precarious employment (PE) trajectories and poor mental health [(SF-12 mental component summary (MSC)]. Regression results are weighted by a longitudinal weight accounting for selective dropout (post-stratification weight*inverse probability weight for selection into analysis sample including education, age, sex, migrant status and occupation as predictors); P< 0.05 was regarded as statistically significant **Bold signifies statistical significance.** [OR=odds ratio; CI=confidence interval.]

		Unstratified sample ^a^ (N=1636)					Women ^b^ (N=905)					Men ^b^ (N=731)			
		Total N	Cases t_0_ N	Cases t_3_ N	OR (95% CI)		Total N	Cases t_0_ N	Cases t_3_ N	OR (95% CI)		Total N	Cases t_0_ N	Cases t_3_ N	OR (95% CI)
MCS cut-off at 47.0 ^c^ (at t_0_ 25% of sample below)															
	Constant non-PE (reference)	814	201	245	1		331	93	109	1		483	108	136	1
	Constant borderline PE	599	136	195	1.00 (0.74–1.35)		391	98	143	1.21 (0.84–1.75)		208	38	52	0.87 (0.53–1.43)
	PE with upward movement	223	62	87	1.22 (0.80–1.86)		183	54	79	**1.68 (1.06–2.66)**		40	8	8	**0.37 (0.14–0.94)**
	Pseudo R^2^				0.079					0.093					0.105
MCS cut-off at 45.6 ^c^ (at t_0_ 21% of sample below)															
	Constant non-PE (reference)	814	166	216	1		331	76	97	1		483	90	119	1
	Constant borderline PE	599	121	173	1.04 (0.77–1.40)		391	91	128	1.17 (0.81–1.69)		208	30	45	0.97 (0.59–1.60)
	PE with upward movement	223	55	81	1.37 (0.90–2.09)		183	47	73	**1.78 (1.12–2.82)**		40	8	8	0.43 (0.16–1.11)
	Pseudo R^2^				0.080					0.077					0.111
MCS cut-off at 42.0 ^c^ (at t_0_ 14% of sample below)															
	Constant non-PE (reference)	814	107	151	1		331	52	69	1		483	55	82	1
	Constant borderline PE	599	90	120	0.97 (0.69–1.36)		391	68	92	1.10 (0.74–1.65)		208	22	28	0.80 (0.43–1.50)
	PE with upward movement	223	36	59	1.47 (0.92–2.34)		183	31	53	**1.82 (1.11–3.02)**		40	5	6	0.51 (0.18–1.44)
	Pseudo R^2^				0.079					0.057					0.145

^a^ Adjusted for sex, age, education, migrant status, partner status, occupation, and mental health status at baseline (t_0_). ^b^ Adjusted for age, education, migrant status, partner status, occupation, and mental health status at baseline (t_0_). ^c^ Values of equal or below indicate poor mental health.

In the sex-stratified analysis (table 2), using the cut-off 47.0, we found that the OR of reporting poor mental health at t_3_ was 1.68 (95% CI 1.06–2.66) for women following a PE trajectory with upward movement compared to women following a constant non-PE trajectory. Using cut-offs 45.6 and 42.0, the OR increased to 1.78 (95% CI 1.12–2.82) and 1.82 (95% CI 1.11–3.02) respectively. Among men the OR was 0.37 (95% CI 0.14–0.94) when using the cut-off 47.0. Using cut-offs 45.6 and 42.0 for SF-12 MCS, this association slightly increased to 0.43 (95% CI 0.16–1.11) and 0.51 (95% CI 0.18–1.44), respectively, and lost statistical significance. No statistically significant longitudinal association between the constant borderline PE trajectory and poor mental health was found.

### Sensitivity analysis

Repeating the logistic regression without a sample weight (supplementary table S10) we found no statistically significant association in the unstratified sample and among men in the sex-stratified sample. Furthermore, weighted and unweighted linear regression (supplementary table S11) showed no statistically significant associations. Further descriptive statistics showed a general trend towards improving employment conditions among PE trajectory members (supplementary tables S12 and S13). This improvement was stronger among men, especially with respect to income. When GBTM was conducted stratified by sex (supplementary figures S14 and S15), we found very similar patterns. Allowing for up to two unemployment spells (supplementary material S16-S21) resulted in similarly shaped trajectories, with the 4-group option with linear terms as the only one fulfilling all the selection criteria described in the method section. In the logistic regression in the unstratified sample, PE with upward movement was associated with increased but non-significant OR to report poor mental health at t_3_. In the analyses stratified by sex, OR was significant among women (1.65–2.04). Among men, PE with upward movement was associated with lower odds to report poor mental health at t_3_. This was significant using the cut-offs 47.1 (25% percentile) and 45.6 but not with the cut-off 42.0.

## Discussion

The aim of the present study was to investigate the longitudinal association between trajectories of PE and mental health. We identified a PE trajectory with upward movement that best described 13.6% of the study sample. Representation in this group was socially unequally distributed with noticeably larger shares of female, lower-educated and lower-skilled workers in PE.

In the non-stratified analyses, the group of persons following the PE with upward movement trajectory (versus constant non-PE) showed increased odds to report poor mental health at t_3_. This association was non-significant. In the sex-stratified analyses, among women, those following the PE trajectory with upward movement had significantly increased odds to report poor mental health at the last survey (t_3_) (OR1.68–1.82 depending on cut-off level). Among men those following the PE trajectory with upward movement had reduced odds to report poor mental health at t_3_ using the cut-off 47.0 (OR 0.37, 95% CI 0.14–0.94). The association was statistically non-significant when outcome cut-offs were lowered to 45.6 and 42.0 to define more severe mental health cases.

### Comparability with existing evidence

In line with previous studies on the risk of PE for mental health (5, 7, 8, 15, 16, 37), our study finds evidence for the longitudinal association between trajectories in PE and mental health. Rönnblad et al's systematic review and meta-analysis (1) contained five studies using a multidimensional PE measurement, of which two may be comparable to our study with respect to exposure measurement (7, 37). Canivet et al (7) found an incidence ratio of 1.4 (95% CI 1.1–2.0) for poor mental health using exposure data combining unemployment, temporary versus permanent employment and job insecurity. Virtanen et al (37) found an OR of up to 1.67 (95% CI 0.78–3.58) adjusting for unemployment and up to 2.33 (95% CI 0.99–5.51) not adjusting for unemployment for suboptimal mental health combining job insecurity and temporary employment as the exposure. These findings approximate those from our unstratified analyses using the outcome cut-off 42.0. However, both studies investigated a younger sample (mean age at baseline was 27 or 30 years, respectively) and did not provide a sex-stratified analysis. Burr (38) very recently provided an overview over longitudinal studies on the topic and constitutes that most sex-stratified analyses found stronger associations in men compared to women. In our study women in PE with upward movement were more likely and men were less likely to report poor mental health than their counterparts following a non-PE trajectory. We found four plausible explanations for our findings and the differences and similarities to existing studies.

Firstly, within-group differences: The multitude of (sensitivity) analyses revealed that men assigned to the PE-group were less precariously employed than women and most of them may likely experience a maximum of two indicators simultaneously by the end of the observation time. Results from Burr (38) suggest that for single men exposed to only one PE indicator rather than multiple adverse employment conditions, the association between PE and depressive symptoms may be negative (OR 0.50, 95% CI 0.06–4,29) (38). Our sensitivity analyses showed that single men had higher chances than those with a partner to be represented in the PE-group (supplementary table S6), hence our results may point to a comparable phenomenon. Our additional analyses showed that among both sexes the prevalence of many of the PE indicators decreased over time while the share of workers with multiple jobs remained stable (supplementary tables S12 and S13). Previous results from Jonsson et al (5) suggest that multiple job holdings may be mentally hazardous for women but not men. Our additional analysis furthermore showed that employment conditions improved more among men with respect to income (supplementary tables S12 and S13). Previous findings point at this PE component to be a particular risk factor for mental health (16, 38). Reduced odds for men following the PE trajectory compared to men following constant non-PE might be a result of the combination of few adverse employment conditions for men in PE, and assumably higher prevalence of other mental health risk factors in the constant non-PE group (see supplementary table S13).

The second explanation is that the experience of PE depends on context. A recent qualitative study by Lain et al (39) indicated that especially older women reported a heightened perception of precarity due to the interaction of PE conditions, financial insecurities due to repeated absences from the labor market and a resulting lack of choice with regard to the timing of retirement (39). This may also apply to Germany, where women are more likely to be unable to amass sufficient financial resources to have adequate control over their working life due to highly gendered unpaid care duties (9, 40, 41) and the associated higher risk of discontinuous and PE patterns (42). The concomitant mental health consequences may be more evident in women approaching retirement. This may explain the differences in findings compared to studies including younger employees.

A third explanation for the differences in study findings could be that due to the unobserved third dimension of PE, namely "lack of rights and protection" (see 3), some cases within the PE with upward movement trajectory could be misclassified.

The fourth explanation is that an unequal selection into the study sample, resulting in a healthier, less precariously employed population may have shaped the upward movement of the PE trajectory and biased the findings.

### Implications

We infer that – when it comes to older workers – the institutional context in Germany allows for a very selective distribution of labor market risks to the disadvantage of female and lower educated workers and those in non-qualified manual and non-manual occupations. These intra-generational horizontal (by sex) and vertical (by qualification) inequalities regarding the exposure to PE should be reduced. Moreover, we assume that especially among older female workers, the constant exposure to employment insecurity and income inadequacy adds to further mental health risks, such as combining work and family/care duties and fragmented employment biographies, which may aggravate financial insecurities when approaching retirement age (43). This may be very specific to the German contribution-based social security system which heavily links current income and non-employment to (future) welfare support (43, 44). These horizontal (by sex) inequalities regarding the vulnerability to PE should be reduced, eg, by alleviating the impact of temporary work and spells of low earnings on pension eligibility and replacement rates.

### Strengths and limitations

Our study is one of the first German studies to multidimensionally measure PE and to our knowledge, the first German study to use a latent class modelling approach to assess the evolution of PE conditions over time. For our literature search, we applied the same search string used in Rönnblad et al's review (1) to search for all articles published since 4 September 2017, ie, the date of the last search for their review, until 7 February 2023. Our findings were to some extent surprising as we had expected stronger negative associations between PE and mental health among men based on existing evidence. Potential bias associated with selection effects and the trajectory model selection need to be noted.

*Selection effects.* First, we did not exclude subjects with existing poor mental health prior to the outcome assessment to keep sample size large enough to conduct sex stratified analyses. There is a possibility that those with poor health at baseline may select into more PE relations (45). To account for this, we adjusted for the baseline mental health status. Secondly, the prospective design of our study is prone to selection bias, caused by selective unit and item non-response as well as inclusion/exclusion criteria. Initially, we excluded unemployed since we could not separate long- from short-term unemployed. However, previous analyses suggest that short-term spells of unemployment may be indicative for PE (eg 38,). In a sensitivity analysis we therefore allowed for a maximum of two unemployment spells between the first and last waves, which resulted in similar findings. We therefore infer that selective unit non-response (probability of being a panel case) rather than our additional exclusion criteria may bias the findings and contribute to the upward trend of the PE trajectory. The application of a sample-weight does not account for all selection effects but for unequal selection probability by design, unequal participation probability at baseline and for unequal selection into the analysis sample by age, sex, education, migrant status and occupation.

*Model building:* One of our major concerns is that we could not operationalize the third PE dimension "lack of rights and protection" which was suggested by Kreshpaj et al (3). Therefore, we possibly underestimated the association between PE and mental health and/or misclassified some workers. Furthermore, a major pitfall of GBTM is that within-class variance is not accounted for (31). In our case, this may have resulted in a PE group that contains few cases driving the upward movement among both sexes. Consequently, the association between PE and mental health might be underestimated, given that membership in this group may be accompanied by both the adverse consequences of PE and the likely positive consequences of improving PE-components over time. However, the four-group option with linear trajectory shapes was the only model to fulfill all selection criteria.

### Concluding remarks

We found that 13.6% of our sample may be best described as precariously employed with upward movement. Representation in this trajectory group was socially unequally distributed by sex, education and occupation. Very few men followed this trajectory (5.5%) compared to women (20.2%). Women in PE from 2011 to 2022 were much more likely (OR 1.68–1.82) than women in constant non-PE to report poor mental health in 2022. To reduce inequalities in adequate employment conditions, political actions are needed to reduce both, exposure to PE and the vulnerability of those exposed.

## Supplementary material

Supplementary material
