# Supplementary material for: Associations between precarious employment trajectories and mental health among older workers in Germany: Vertical and horizontal inequalities
Source: Scand J Work Environ Health. 2024 Apr 29;50(4):290–9. doi: 10.5271/sjweh.4160 (PMC11130709; doi:10.5271/sjweh.4160)
Supplement: Supplementary material [file SJWEH-50-290-S001.pdf]

Associations between precarious employment trajectories and mental health among older workers in Germany: Vertical and horizontal inequalities[1]

by Max Rohrbacher, MSc,[2] Hans Martin Hasselhorn, MD, Nuria Matilla-Santander, PhD

1. Appendix: Supplementary Figures & Tables

2. Correspondence to: Max Rohrbacher, Department of Occupational Health Science, School of Mechanical Engineering and Safety Engineering, University of Wuppertal, Gaussstrasse 20, 42119 Wuppertal, Germany. [E-mail: rohrbacher@uni-wuppertal.de]. **ORCID:** Max Rohrbacher: **0000-0003-1151-8975;** Hans Martin Hasselhorn: **0000-0002-0317-6218;** Nuria Matilla-Santander: **0000-0002-5249-102X**

Supplementary table S1. Scoring of PE items

Domain	Item	Score				
		-2	-1	0	1	2
Employment insecurity	Job threat	yes		no		
	Temporary employment	temporary		permanent		
	Multiple jobs		≥2 jobs	1 job		
Income inadequacy	Hourly personal net wage	<60%	60-79%	80-99%	100-149%	≥ 150%

Supplementary table S2. Model Selection: Using BIC and AIC to determine number of groups

No. of groups	BIC (N=1 636)	AIC
3	-10614.91	-10590.61
4	-10526.88	-10494.48
5	-10503.09	-10462.59
6	-10485.91	-10437.31

Supplementary table S3. Model Diagnostics of PE trajectories

Group	Estimated proportion from the trajectory model	99% confidence interval for the estimated proportion	Proportion by posterior probability-based classification	Average posterior probability	Odds of correct classification
1	14.63	12.68-16.57	13.63	87.18	39.70
2	33.23	30.90-35.55	36.61	78.03	7.14
3	41.22	38.57-43.88	39.36	89.76	12.50
4	10.92	9.06-12.78	10.39	94.35	136.08

Supplementary figure S4. Directed Acyclic Graph (DAG) (made with DAGitty)

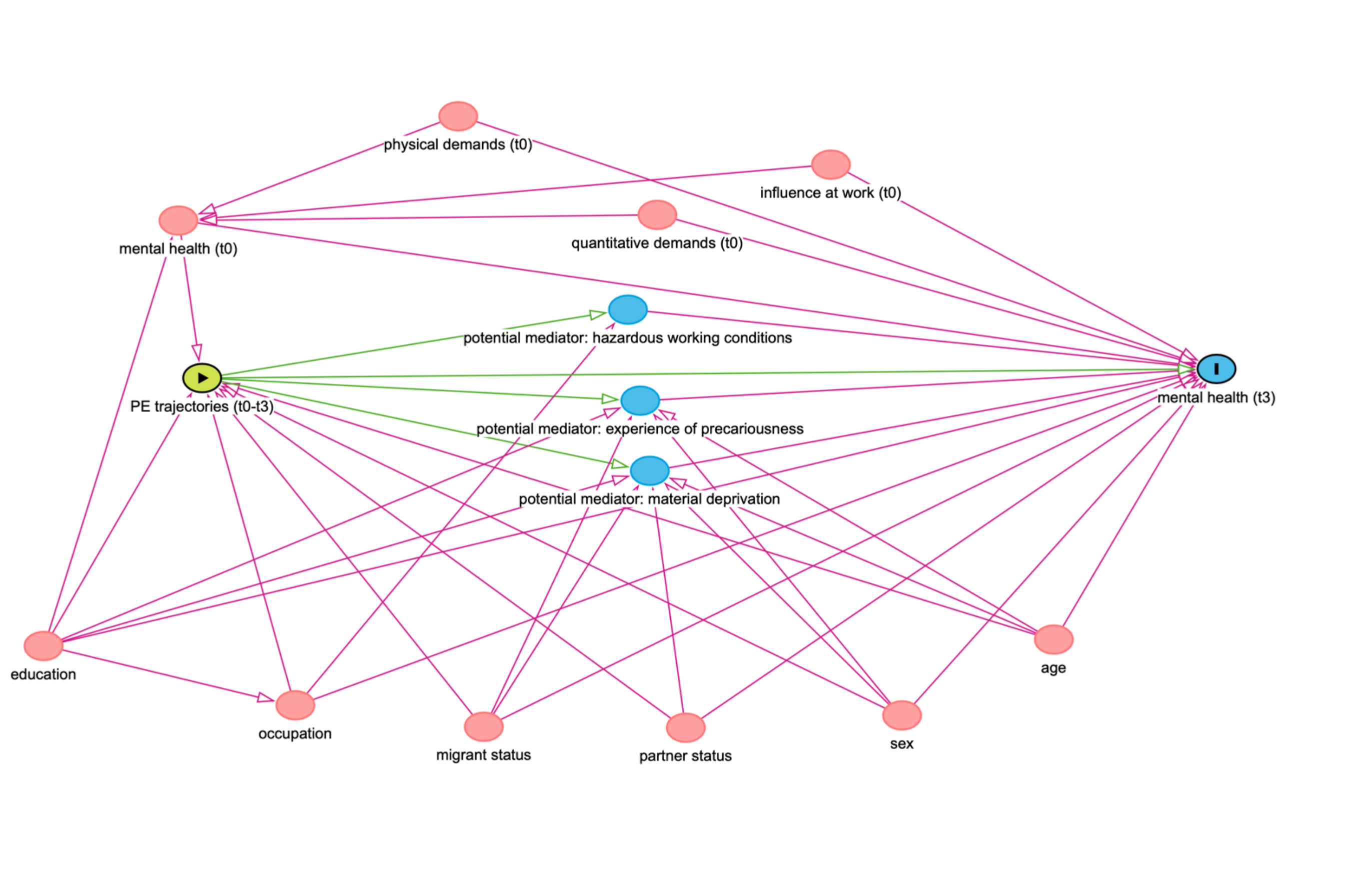

Minimal sufficient adjustment sets for estimating the total effect of PE trajectories (t0-t3) on mental health (t3): age, education, mental health (t0), migrant status, occupation, partner status, sex

Legend: green circle = exposure; blue circle with black line = outcome; blue circles = ancestor of outcome; red circles = ancestor of exposure and outcome; red arrow = biasing path; green arrow = causal path

Supplementary table S5. Sample characteristics of **women** (N=905). Data are presented as **row %**

		**PE with upward movement**	**borderline PE**	**non-PE**
		N=183	N=391	N=331
Age (t_0)	46 (born 1965)	20.9	44.0	35.1
	52 (born 1959)	19.1	42.0	38.9
Migrant	non migrant	19.8	44.2	36.0
	migrant	23.1	36.8	40.2
Partner status (t_0)	with partner	21.4	42.7	35.9
	single	12.6	46.2	41.2
Educational level	Low	30.3	48.5	21.2
	Moderate	20.8	45.7	33.5
	High	11.7	32.5	55.8
Occupation (t_0)	Non-qualified manual	50.0	38.5	11.5
	Qualified manual	21.9	42.2	35.9
	Non-qualified non-manual	45.6	43.5	10.9
	Qualified non-manual	14.4	46.1	39.5
	Highly qualified non-manual	5.9	24.7	69.4
Occupation (t_3)	Non-qualified manual	45.0	45.0	10.0
	Qualified manual	23.3	40.0	36.7
	Non-qualified non-manual	42.8	45.4	11.8
	Qualified non-manual	15.3	45.6	39.1
	Highly qualified non-manual	5.9	24.7	69.4

Authors' summary
Among women the following characteristics are accompanied with higher chances of representation in the "PE with upward movement": younger, migrant background, with partner, low education, non-qualified (non-)manual occupations

Supplementary table S6. Sample characteristics of **men** (N=731). Data are presented as **row %**

		PE with upward movement	borderline PE	non-PE
		**N=40**	**N=208**	**N=483**
Age (t_0)	46 (born 1965)	*	*	68.0
	52 (born 1959)	*	*	62.5
Migrant	non migrant	*	*	65.6
	migrant	*	*	69.2
Partner status (t_0)	with partner	*	*	68.4
	single	12.5	43.1	44.4
Educational level	Low	*	*	48.7
	Moderate	*	*	62.6
	High	*	*	84.6
Occupation (t_0)	Non-qualified manual	9.4	48.2	42.4
	Qualified manual	*	*	63.5
	Non-qualified non-manual	14.9	59.6	25.5
	Qualified non-manual			77.2
	Highly qualified non-manual	*	*	91.3
Occupation (t_3)	Non-qualified manual	9.8	45.1	45.1
	Qualified manual			62.9
	Non-qualified non-manual	14.2	57.5	28.3
	Qualified non-manual	*	*	76.5
	Highly qualified non-manual	*	*	94.7

*This data cannot be shown for reasons of data protection

Authors' summary

Among men the following characteristics are accompanied with higher chances of representation in the "PE with upward movement": older, migrant background, single, low education, non-qualified (non-)manual occupations

Supplementary table S7. Sample characteristics. Data are presented as **column %**

		PE with upward movement	borderline PE	non-PE
		**N=223**	**N=599**	**N=814**
Age (t_0)	46 (born 1965)	137 (61.4%)	374 (62.4%)	516 (63.4%)
	52 (born 1959)	86 (38.6%)	225 (37.6%)	298 (36.6%)
Sex	male	40 (17.9%)	208 (34.7%)	483 (59.3%)
	Female	183 (82.1%)	391 (65.3%)	331 (40.7%)
Migrant	non migrant	188 (84.3%)	532 (88.8%)	695 (85.4%)
	Migrant	35 (15.7%)	67 (11.2%)	119 (14.6%)
Educational level	low	51 (22.9%)	133 (22.2%)	104 (12.8%)
	moderate	140 (62.8%)	378 (63.1%)	419 (51.5%)
	High	32 (14.3%)	88 (14.7%)	291 (35.7%)
Occupation (t_0)	Non-qualified manual	21 (9.4%)	51 (8.5%)	39 (4.8%)
	Qualified manual	24 (10.8%)	90 (15.0%)	150 (18.4%)
	Non-qualified non-manual	81 (36.3%)	120 (20.0%)	40 (4.9%)
	Qualified non-manual	88 (39.5%)	306 (51.1%)	369 (45.3%)
	Highly qualified non-manual	9 (4.0%)	32 (5.3%)	216 (26.5%)
Occupation (t_3)	Non-qualified manual	17 (7.6%)	46 (7.7%)	39 (4.8%)
	Qualified manual	24 (10.8%)	87 (14.5%)	146 (17.9%)
	Non-qualified non-manual	80 (35.9%)	130 (21.7%)	48 (5.9%)
	Qualified non-manual	96 (43.0%)	308 (51.4%)	380 (46.7%)
	Highly qualified non-manual	6 (2.7%)	28 (4.7%)	201 (24.7%)
Mental health cut-off 47.0 (t_0)	poor	62 (27.8%)	136 (22.7%)	201 (24.7%)
Mental health cut-off 47.0 (t_3)	poor	87 (39.0%)	195 (32.6%)	245 (30.1%)
Mental health cut-off 45.6 (t_0)	poor	55 (24.7%)	121 (20.2%)	166 (20.4%)
Mental health cut-off 45.6 (t_3)	poor	81 (36.3%)	173 (28.9%)	216 (26.5%)
Mental health cut-off 42.0 (t_0)	poor	36 (16.1%)	90 (15.0%)	107 (13.1%)
Mental health cut-off 42.0 (t_3)	poor	59 (26.5%)	120 (20.0%)	151 (18.6%)

Supplementary table S8. Longitudinal weight: mean weighting factors by sex and education

	Education		
**Sex**	low	moderate	high
male	1.2979244	.83404445	.66740059
female	1.1827268	.76190257	.68204031

Supplementary table S9. Longitudinal weight: mean weighting factors by sex and occupation (t3)

	Occupation				
**Sex**	Non-qualified manual	Qualified manual	Non-qualified non-manual	Qualified non-manual	Highly qualified non-manual
male	.93757945	.95406273	1.2425496	.80625481	.61214599
female	1.3200514	.71973646	.98978907	.77249671	.6479592

Supplementary table S10. Longitudinal association between precarious work trajectories and mental health (SF-12 MCS). Logistic Regression (unweighted)

	Unstratified sample (n=1636)		Women (n=905)		Men (n=731)	
**MCS cut-off at 47.0**	**N**	**OR (95% CI)**	**N**	**OR (95% CI)**	**N**	**OR (95% CI)**
Constant non-PE (reference)	814	1	331	1	483	1
Constant borderline PE	599	1.02 (0.78-1.32)	391	1.23 (0.88-1.72)	208	0.85 (0.55-1.32)
PE with upward movement	223	1.19 (0.82-1.70)	183	1.50 (0.98-2.29)	40	0.63 (0.26-1.54)
Pseudo R^2		0.078		0.074		0.089
**MCS cut-off at 45.6**		**OR (95% CI)**		**OR (95% CI)**		**OR (95% CI)**
Constant non-PE (reference)	814	1	331	1	483	1
Constant borderline PE	599	0.98 (0.75-1.27)	391	1.14 (0.78-1.66)	208	0.68 (0.39-1.18)
PE with upward movement	223	1.24 (0.86-1.78)	183	1.58 (0.99-2.52)	40	0.78 (0.29-2.13)
Pseudo R^2		0.068		0.045		0.086
**MCS cut-off at 42.0**		**OR (95% CI)**		**OR (95% CI)**		**OR (95% CI)**
Constant non-PE (reference)	814	1	331	1	483	1
Constant borderline PE	599	0.93 (0.69-1.26)	391	1.17 (0.83-1.64)	208	0.82 (0.52-1.29)
PE with upward movement	223	1.28 (0.86-1.91)	183	**1.54 (1.00-2.37)**	40	0.69 (0.28-1.70)
Pseudo R^2		0.061		0.059		0.082
**Adjusted** for sex (unstratified sample only), age, education, migrant status, partner status, occupation, and mental health status at baseline (t_0)<br>[a]Values of equal or below indicate poor mental health						

Supplementary table S11. Linear Regression (weighted and unweighted)

	Unstratified sample (n=1636)	Women (n=905)	Men (n=731)
**Weighted Linear Regression (MCS Score 0-100)** (higher values indicate better mental health)	**Beta (95% CI)**	**Beta (95% CI)**	**Beta (95 % CI)**
Constant non-PE (reference)	1	1	1
Constant borderline PE	0.33 (-0.87; 1.52)	-0.20 (-1.65; 1.61)	0.16 (-1.55; 1.87)
PE (with upward movement)	-0.85 (-2.63; 0.92)	-1.57 (-3.65; 0.51)	1.92 (-1.38; 5.24)
R^2	0.18	0.17	0.209
**Unweighted Linear Regression (MCS Score 0-100)** (higher values indicate better mental health)	**Beta (95% CI)**	**Beta (95% CI)**	**Beta (95 % CI)**
Constant non-PE (reference)	1	1	1
Constant borderline PE	0.33 (-0.76; 1.43)	-0.31 (-1.84; 1.21)	0.72 (-0.88; 2.31)
PE (with upward movement)	-0.77 (-2.35; 0.80)	-1.52 (-3.50; 0.47)	1.32 (-1.66; 4.30)
R^2	0.15	0.135	0.167
**Adjusted** for sex (unstratified sample only), age, education, migrant status, partner status, occupation, and mental health status at baseline (t_0)<br>P< 0.05 was regarded as statistically significant			

Supplementary table S12. Female sub-sample: PE components over time, work factors and occupations. Data are presented as **column %** or M(SD)

		PE with upward movement	borderline PE	non-PE
		N=183	N=391	N=331
		% or M(SD)	% or M(SD)	% or M(SD)
temporary employment (t_0)	temporary	21.9	3.6	*
temporary employment (t_1)	temporary	15.8	2.0	*
temporary employment (t_2)	temporary	12.0	1.5	*
temporary employment (t_3)	temporary	6.0	1.5	*
hourly personal net income (t_0)	<60%	57.9	14.1	*
	60-79%	28.4	34.0	3.6
	80-99%	9.8	31.2	18.1
	100-149%	3.8	17.6	56.8
	≥ 150%	0.0	3.1	20.5
hourly personal net income (t_1)	<60%	57.9	13.3	*
	60-79%	29.0	28.1	*
	80-99%	7.7	40.4	19.6
	100-149%	*	15.6	57.4
	≥ 150%	*	2.6	20.5
hourly personal net income (t_2)	<60%	61.7	15.3	*
	60-79%	26.2	32.2	*
	80-99%	*	34.5	17.8
	100-149%	*	15.9	57.7
	≥ 150%	*	2.0	21.1
hourly personal net income (t_3)	<60%	44.8	9.2	*
	60-79%	38.3	37.3	*
	80-99%	14.8	34.3	17.2
	100-149%	*	17.4	58.9
	≥ 150%	*	1.8	21.1
job threat (t_0)	yes	23.0	5.9	*
job threat (t_1)	yes	15.3	6.9	2.1
job threat (t_2)	yes	13.1	6.6	3.0
job threat (t_3)	yes	8.7	5.1	*
multiple jobs (t_0)	yes	26.8	12.0	6.3
multiple jobs (t_1)	yes	26.8	11.3	6.3
multiple jobs (t_2)	yes	27.3	14.1	6.0
multiple jobs (t_3)	yes	23.0	11.8	5.4
physical demands (t_0)	often/ ≥25% of time	58.5	54.5	45.0
physical demands (t_1)	often/ ≥25% of time	53.0	51.7	43.5
physical demands (t_2)	often/ ≥25% of time	58.5	58.3	50.2
physical demands (t_3)	often/ ≥25% of time	59.6	57.5	48.3
quantitative demands (t_0)		40.75 (25.46)	45.60 (23.30)	48.45 (22.77)
quantitative demands (t_1)		39.29 (22.80)	45.63 (23.85)	49.87 (22.03)
quantitative demands (t_2)		39.79 (22.25)	46.37 (20.63)	48.91 (21.49)
quantitative demands (t_3)		39.93 (21.52)	47.16 (22.15)	49.16 (20.65)
influence at work (t_0)		29.87 (27.01)	33.87 (26.97)	41.04 (24.79)
influence at work (t_1)		34.26 (26.88)	35.97 (26.22)	40.53 (23.90)
influence at work (t_2)		29.53 (24.57)	32.18 (25.33)	38.54 (24.18)
influence at work (t_3)		29.37 (25.98)	33.22 (26.18)	38.50 (23.85)
Occupation (t_0)	Non-qualified manual	7.1	2.6	*
	Qualified manual	7.7	6.9	6.9
	Non-qualified non-manual	36.6	16.4	*
	Qualified non-manual	45.9	68.8	69.5
	Highly qualified non-manual	2.7	5.4	17.8
Occupation (t_3)	Non-qualified manual	4.9	2.3	*
	Qualified manual	7.7	6.1	6.6
	Non-qualified non-manual	35.5	17.6	*

	Qualified non-manual	49.2	68.5	69.5
	Highly qualified non-manual	2.7	5.4	17.8
occupational sectors (t_0)	Production	7.1	5.4	6.3
	Personal services	39.9	40.4	45.3
	Commercial and business service	42.1	46.3	39.9
	IT and science service	*	2.0	6.6
	Other economic services	*	5.9	1.8
occupational sectors (t_3)	Production	5.5	5.6	6.9
	Personal services	42.1	42.5	43.8
	Commercial and business service	39.3	45.5	40.8
	IT and science service	*	*	6.3
	Other economic services	*	*	2.1

*This data cannot be shown for reasons of data protection

Authors' summary:
Main differences between women and men (see **S13**) are observed for changes in income, working conditions, occupation and occupational sector. The ***characteristics of women*** *in "PE with upward movement"* can be described as follows:

- **Income:** marginal changes; share of those with >100% income reduces over time; share of those with >80% is fairly stable (t0-t3: 13.6%, 13.1%, 12%, 16.9%) - increase at t3 is likely a result of decreasing share with >100% income; share with <80% income increasing until t_2
- **Working conditions:** slight increase of share who is often exposed to physical demands; mean quantitative demands and influence at work are stable
- **Occupations:** about 80% are in either personal service occupations or commercial and business service occupations, high share of non-manual occupations (non-qualified and qualified), women in PE work mostly in either small (>50 employees) or very large firms (>1000 employees) (not displayed)

Supplementary table S13. Male sub-sample: PE components over time, work factors and occupations. Data are presented as **column %** or M(SD)

		PE with upward movement	borderline PE	non-PE
		N=40	N=208	N=483
		% or M(SD)	% or M(SD)	% or M(SD)
w1 temporary employment	temporary	17.5	4.8	0.2
w2 temporary employment	temporary	15.0	4.3	*
w3 temporary employment	temporary	10.0	2.4	0.2
w4 temporary employment	temporary	*	*	0.4
w1 hourly personal income	<60%	47.5	4.3	*
	60-79%	37.5	37.5	0.8
	80-99%	*	34.6	10.1
	100-149%	*	20.7	41.4
	≥ 150%	*	2.9	47.6
w2 hourly personal income	<60%	37.5	5.3	0.2
	60-79%	45.0	22.6	1.0
	80-99%	*	51.4	8.9
	100-149%	*	18.3	47.2
	≥ 150%	*	2.4	42.7
w3 hourly personal income	<60%	47.5	9.1	0.4
	60-79%	27.5	28.8	1.2
	80-99%	*	38.0	11.0
	100-149%	*	21.6	42.7
	≥ 150%	*	2.4	44.7
w4 hourly personal income	<60%	45.0	5.3	*
	60-79%	30.0	32.2	*
	80-99%	12.5	38.9	11.2
	100-149%	*	*	46.8
	≥ 150%	*	*	39.8
w1 job threat	yes	32.5	11.1	5.4
w2 job threat	yes	27.5	11.1	5.2
w3 job threat	yes	15.0	7.2	3.7
w4 job threat	yes	12.5	10.6	3.1
w1 multiple jobs	yes	17.5	13.9	7.7
w2 multiple jobs	yes	22.5	14.4	7.7

w3 multiple jobs	yes	20.0	15.4	7.0
w4 multiple jobs	yes	20.0	13.9	7.7
physical demands (t_0)	often/ ≥25% of time	60.0	56.7	41.2
physical demands (t_1)	often/ ≥25% of time	57.5	55.3	37.5
physical demands (t_2)	often/ ≥25% of time	57.5	50.5	40.4
physical demands (t_3)	often/ ≥25% of time	52.5	56.3	42.4
quantitative demands (t_0)		44.37 (22.27)	41.80 (22.11)	49.90 (21.17)
quantitative demands (t_1)		43.33 (20.60)	40.54 (21.09)	49.49 (22.18)
quantitative demands (t_2)		45.83 (21.84)	41.74 (19.03)	49.36 (20.09)
quantitative demands (t_3)		39.16 (24.83)	41.26 (20.81)	47.67 (21.25)
influence at work (t_0)		33.33 (25.59)	36.29 (27.44)	47.04 (24.86)
influence at work (t_1)		33.54 (24.56)	36.01 (28.57)	47.43 (24.51)
influence at work (t_2)		32.5 (25.72)	36.49 (27.13)	43.22 (23.54)
influence at work (t_3)		40.83 (30.06)	37.541 (26.30)	44.43 (25.01)
Occupation (t_0)	Non-qualified manual	20.0	19.7	7.5
	Qualified manual	25.0	30.3	26.3
	Non-qualified non-manual	35.0	26.9	5.0
	Qualified non-manual	10.0	17.8	28.8
	Highly qualified non-manual	20.0	19.7	7.5
Occupation (t_3)	Non-qualified manual	20.0	17.8	7.7
	Qualified manual	25.0	30.3	25.7
	Non-qualified non-manual	37.5	29.3	6.2
	Qualified non-manual	*	19.2	31.1
	Highly qualified non-manual	*	3.4	29.4
occupational sectors (t_0)	Production	42.5	55.3	42.7
	Personal services	12.5	10.1	13.7
	Commercial and business service	12.5	14.9	30.2
	IT and science service	*	1.4	7.9
	Other economic services	*	18.3	5.6
occupational sectors (t_3)	Production	45.0	51.9	41.0
	Personal services	17.5	10.6	13.5
	Commercial and business service	*	14.9	30.0
	IT and science service	*	2.9	8.7
	Other economic services	*	19.7	6.8

*This data cannot be shown for reasons of data protection

Authors' summary:

Main differences between women (see **S12**) **and men** are observed for changes in income, working conditions, occupation and occupational sector. The ***characteristics of men*** *in "PE with upward movement"* can be described as follows:

- **Income:** growing share of those with >80% income (t0-t3: 15%, 17.5%, 25%, 25%)
- **Working conditions:** slight decrease of share who is often exposed to physical demands; mean quantitative demands decrease over time; mean influence at work strongly improves
- **Occupations:** about 75% are in "production" (40-45%) or "other economic services"; high share of manual (non-qualified and qualified) and non-qualified non-manual occupations; men in PE work with almost equal share in all sizes of firms with slightly higher share in medium size firms (not displayed)

Supplementary figure S14. PE trajectories modelled in **female** sub-sample (% = proportion by posterior probability-based classification).

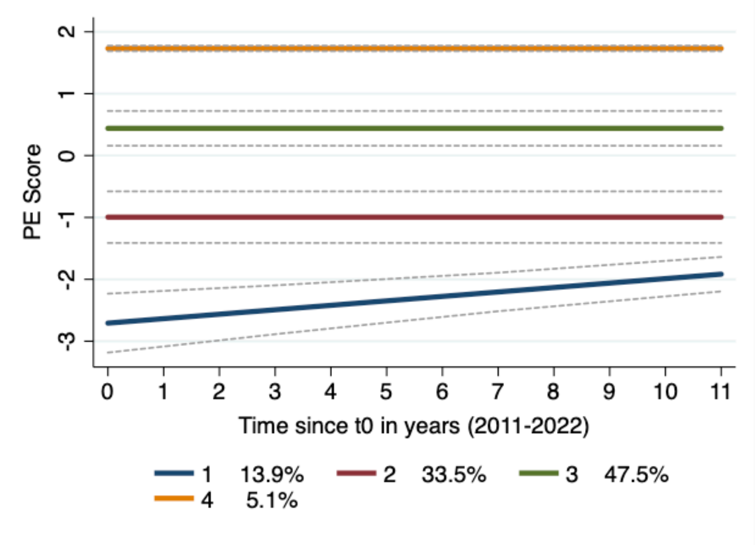

Supplementary figure S15. PE trajectories modelled in **male** sub-sample (% = proportion by posterior probability-based classification).

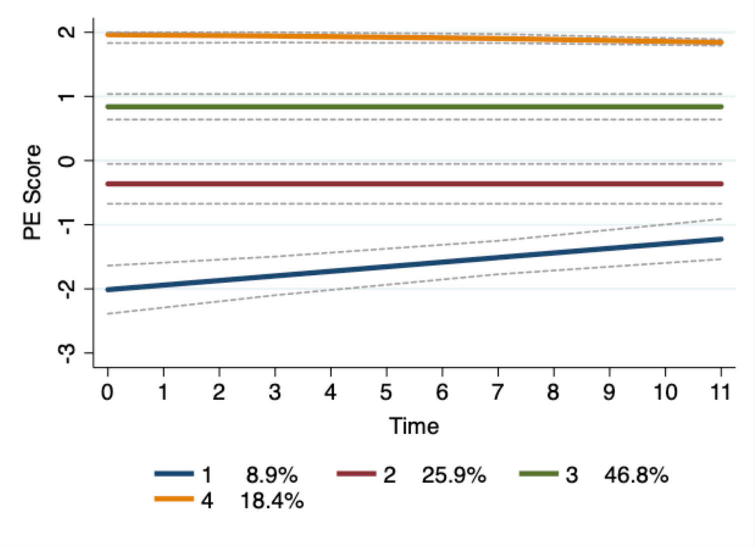

Supplementary figure S16. Flow chart of inclusion and exclusion criteria (unemployment at t_1 and t_2 allowed)

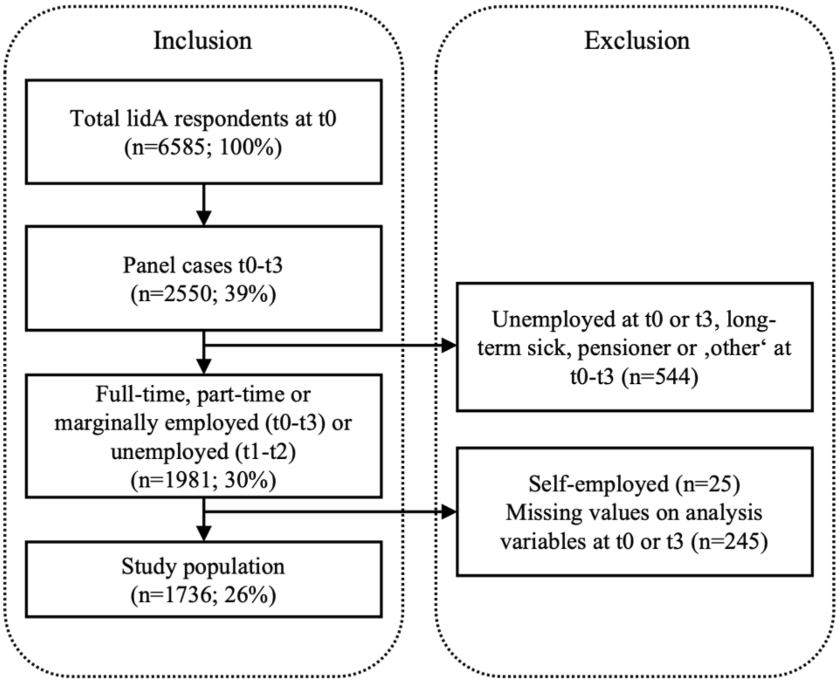

Supplementary table S17. Model Selection: Using BIC and AIC to determine number of groups (unemployment at t_1 and t_2 allowed)

No. of groups	BIC (N=1 736)	AIC
3	-11290.54	-11265.98
4	-11209.05	-11176.30
5	-11187.49	-11146.55
6	-11171.87	-11122.74

Supplementary table S18. Model Diagnostics of PE trajectories (unemployment at t_1 and t_2 allowed)

Group	Estimated proportion from the trajectory model	99% confidence interval for the estimated proportion	Proportion by posterior probability-based classification	Average posterior probability	Odds of correct classification
1	13.61	11.80-15.42	13.02	84.95	37.71
2	32.21	29.99-34.43	30.76	81.81	10.12
3	42.98	40.40-45.57	45.10	86.56	7.84
4	11.19	9.40-13.01	11.12	91.97	91.58

Supplementary figure S19. PE Trajectories of precarious and non-precarious employment (N=1736). An individual was regarded as precariously employed when the PE Score was -2 and below (% = proportion by posterior probability-based classification). Measurements took place in 2011, 2014, 2018, 2022. (unemployment at t_1 and t_2 allowed)

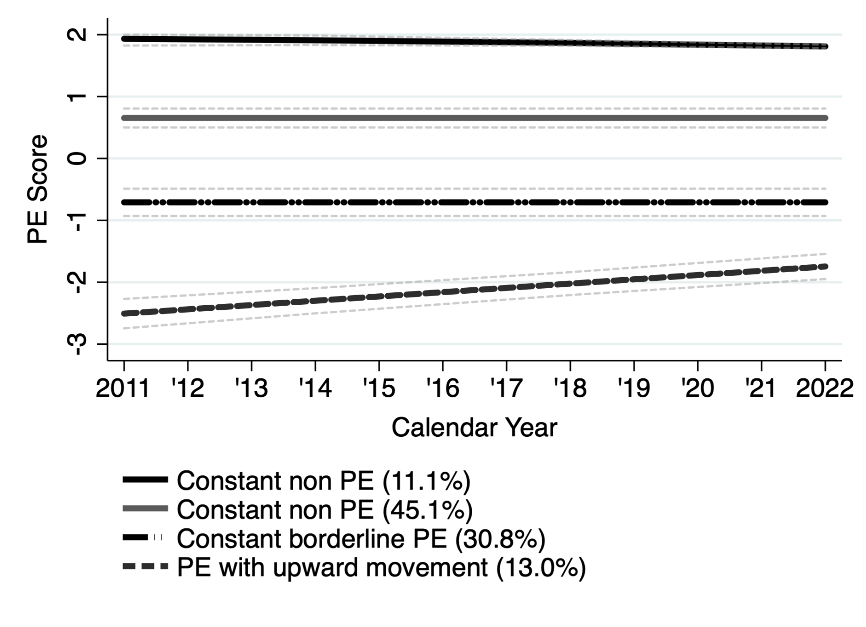

Supplementary table S20. Sample characteristics. Data are presented as **row %** (unemployment at t_1 and t_2 allowed).

		PE with upward movement N=226	borderline PE N=534	non-PE N=976
**Age (t_0)**	46 (born 1965)	141 (13.0%)	332 (30.6%)	612 (56.4%)
	52 (born 1959)	85 (13.1%)	202 (31.0%)	364 (55.9%)
**Sex**	Male	39 (5.0%)	181 (23.3%)	556 (71.6%)
	Female	187 (19.5%)	353 (36.8%)	420 (43.8%)
**Migrant**	Non migrant	195 (13.0%)	470 (31.4%)	830 (55.5%)
	Migrant	31 (12.9%)	64 (26.6%)	146 (60.6%)
**Educational level**	Low	52 (16.9%)	122 (39.6%)	134 (43.5%)
	Moderate	143 (14.4%)	335 (33.7%)	517 (52.0%)
	High	31 (7.2%)	77 (17.8%)	325 (75.1%)
**Occupation (t_0)**	Simple manual	23 (19.0%)	42 (34.7%)	56 (46.3%)
	Qualified manual	19 (6.9%)	79 (28.7%)	177 (64.4%)
	Simple non-manual	83 (32.5%)	118 (46.3%)	54 (21.2%)
	Qualified non-manual	90 (11.1%)	268 (32.9%)	456 (56.0%)
	Highly qualified non-manual	11 (4.1%)	27 (10.0%)	233 (86.0%)
**Occupation (t_3)**	Simple manual	21 (18.9%)	35 (31.5%)	55 (49.5%)
	Qualified manual	18 (6.7%)	79 (29.5%)	171 (63.8%)
	Simple non-manual	83 (29.3%)	131 (46.3%)	69 (24.4%)
	Qualified non-manual	96 (11.6%)	267 (32.2%)	465 (56.2%)
	Highly qualified non-manual	8 (3.3%)	22 (8.9%)	216 (87.8%)
**Unemployment (t_1)**	No	216 (12.6%)	526 (30.7%)	974 (56.8%)
	Unemployed (N= 20)	10 (50.0%)	*	*
**Unemployment (t_2)**	No	221 (12.9%)	526 (30.6%)	970 (56.5%)
	Unemployed (N=19)	5 (26.3%)	8 (42.1%)	6 (31.6%)

*This data cannot be shown for reasons of data protection

Supplementary table S21. Longitudinal association between precarious work trajectories and poor mental health (SF-12 MCS). Logistic Regression (weighted). Statistically significant ORs and 95% CIs in bold. (unemployment at t_1 and t_2 allowed)

	Unstratified sample (n=1736)		Women (n=960)		Men (n=776)	
**MCS cut-off at 47.1[a]**	**N**	**OR (95% CI)**	**N**	**OR (95% CI)**	**N**	**OR (95% CI)**
Constant non-PE (reference)	226	1	420	1	556	1
Constant borderline PE	534	0.95 (0.71-1.27)	353	1.32 (0.92-1.88)	281	0.65 (0.39-1.09)
PE with upward movement	976	1.15 (0.76-1.73)	187	**1.65 (1.06-2.57)**	39	**0.33 (0.13-0.83)**
Pseudo R^2		0.076		0.085		0.104
**MCS cut-off at 45.6[a]**		**OR (95% CI)**		**OR (95% CI)**		**OR (95% CI)**
Constant non-PE (reference)	226	1	420	1	556	1
Constant borderline PE	534	1.08 (0.81-1.44)	353	**1.51 (1.06-2.16)**	281	0.68 (0.40-1.16)
PE with upward movement	976	1.38 (0.91-2.10)	187	**2.04 (1.31-3.17)**	39	**0.37 (0.14-0.97)**
Pseudo R^2		0.080		0.077		0.119
**MCS cut-off at 42.0[a]**		**OR (95% CI)**		**OR (95% CI)**		**OR (95% CI)**
Constant non-PE (reference)	226	1	420	1	556	1
Constant borderline PE	534	0.96 (0.69-1.34)	353	1.36 (0.92-2.00)	281	**0.50 (0.25-0.97)**
PE with upward movement	976	1.40 (0.89-2.21)	187	**1.91 (1.18-3.07)**	39	0.48 (0.18-1.33)
Pseudo R^2		0.073		0.051		0.139

Adjusted for sex (unstratified sample only), age, education, migrant status, partner status, occupation, and mental health status at baseline (t_0)

[a]Values of equal or below indicate poor mental health

Regression results are weighted by a longitudinal weight accounting for selective dropout (post-stratification weight*inverse probability weight for selection into analysis sample including education, age, sex, migrant status and occupation as predictors)

P< 0.05 was regarded as statistically significant
